# Sphingolipids in thyroid eye disease

**DOI:** 10.3389/fendo.2023.1170884

**Published:** 2023-04-04

**Authors:** Anne Gulbins, Gina-Eva Görtz, Erich Gulbins, Anja Eckstein

**Affiliations:** ^1^Department of Ophthalmology, University Hospital Essen, University of Duisburg-Essen, Essen, Germany; ^2^Institute of Molecular Biology, University Hospital Essen, University of Duisburg-Essen, Essen, Germany

**Keywords:** thyroid eye disease (TED), Graves’ disease, sphingolipids, ceramide, acid sphingomyelinase 2

## Abstract

Graves’ disease (GD) is caused by an autoimmune formation of autoantibodies and autoreactive T-cells against the thyroid stimulating hormone receptor (TSHR). The autoimmune reaction does not only lead to overstimulation of the thyroid gland, but very often also to an immune reaction against antigens within the orbital tissue leading to thyroid eye disease, which is characterized by activation of orbital fibroblasts, orbital generation of adipocytes and myofibroblasts and increased hyaluronan production in the orbit. Thyroid eye disease is the most common extra-thyroidal manifestation of the autoimmune Graves’ disease. Several studies indicate an important role of sphingolipids, in particular the acid sphingomyelinase/ceramide system and sphingosine 1-phosphate in thyroid eye disease. Here, we discuss how the biophysical properties of sphingolipids contribute to cell signaling, in particular in the context of thyroid eye disease. We further review the role of the acid sphingomyelinase/ceramide system in autoimmune diseases and its function in T lymphocytes to provide some novel hypotheses for the pathogenesis of thyroid eye disease and potentially allowing the development of novel treatments.

## 1 Introduction

**Figure 1:**
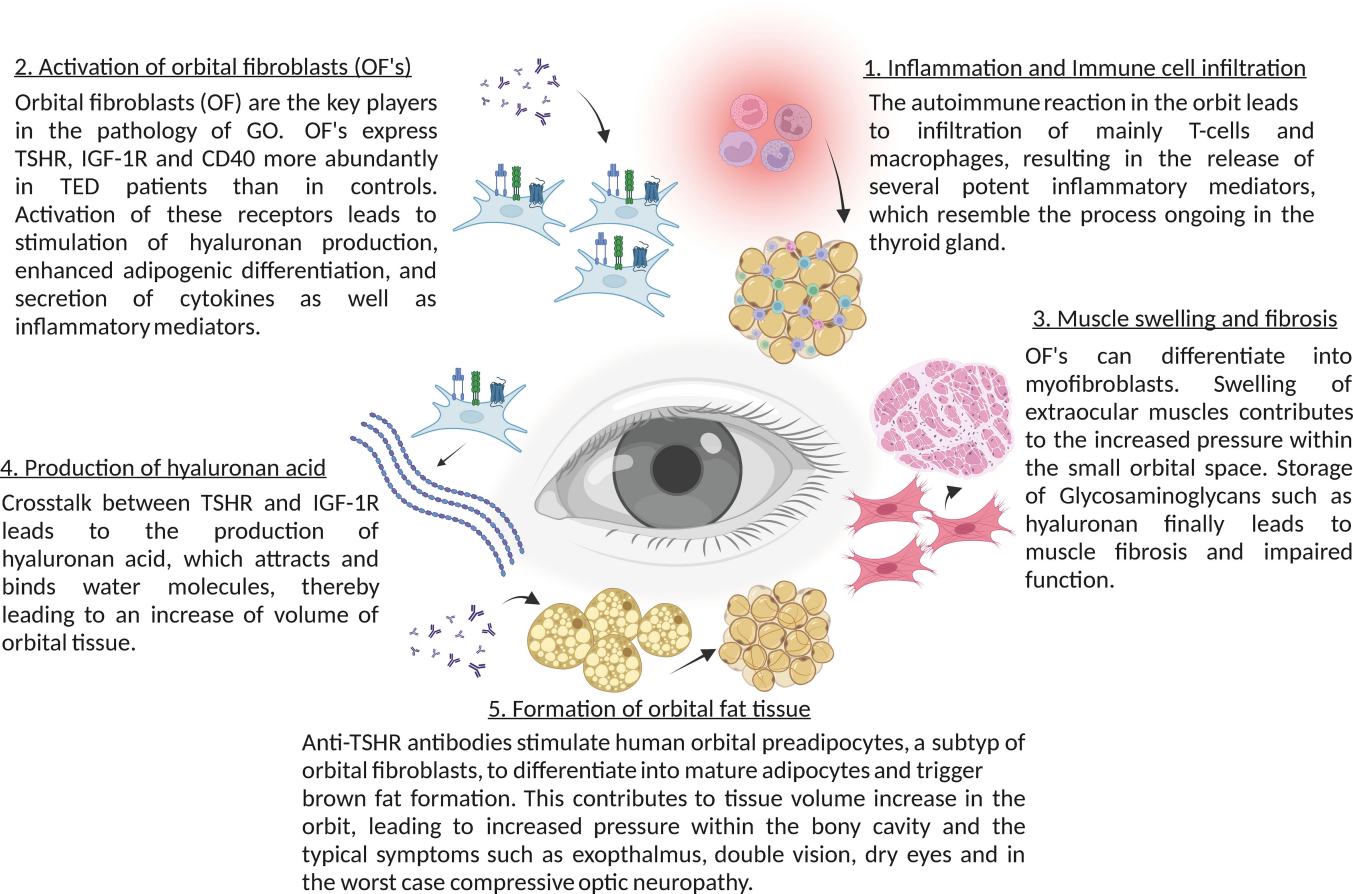
Summary of the main molecular mechanisms in thyroid eye disease (TED).

**Figure 2:**
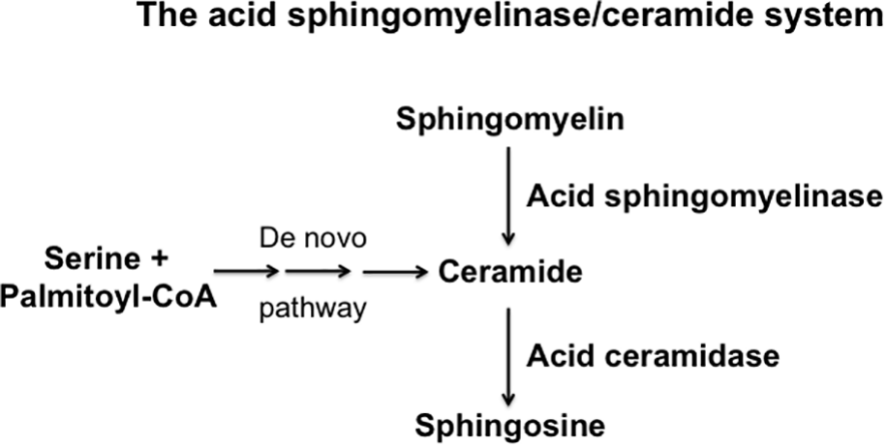
Schematic overview over the synthesis and consumption of ceramide, sphingomelin and sphingosine.

Sphingolipids have been shown to be important in several autoimmune diseases such as multiple sclerosis and arthritis (1–4). Both, the *de novo* synthesis of sphingolipids and the sphingomyelinase-pathway are implied in the pathogenesis of auto-immune disorders (1–4). However, at present only limited information is available on the role of sphingolipids in Graves’ disease (GD) and thyroid eye disease. GD is an autoimmune disorder, which is caused by activation of the immune system against the endogenous thyroid stimulating hormone receptor (TSHR), with the formation of auto-antibodies that trigger alterations and the clinical symptoms affecting the thyroid, the eye orbit and other tissues. Here, we summarize the current knowledge about the role of sphingolipids in autoimmune diseases, in particular GD, present an overview about the pathophysiology of GD and thyroid eye disease and discuss hypotheses how to combine the two areas of research. The current knowledge on thyroid eye disease is briefly summarized in **Figure 1** (please refer for an extended presentation to the chapters below). The pathways of ceramide synthesis and sphingomyelinase-activities are given in **Figure 2**.

## 2 Sphingolipids and ceramide

Sphingolipids belong to the major components of the biological membranes. They are composed of the very hydrophobic ceramide moiety and a hydrophilic headgroup, for instance phosphorylcholine for the abundant membrane lipid sphingomyelin. Sphingolipids are critically involved in the structure of cellular membranes (5, 6). However, they are not only structural molecules of cellular membranes, but also involved in cellular signal transduction, cell stimulation, stress and death. Sphingomyelin molecules interact with each other and together with cholesterol molecules that stabilize the bulky sphingomyelin aggregates, they contribute to the organization of cellular membranes and the formation of small distinct membrane domains (5–7). These very small domains, with a diameter of approximately 20 nm (8), were named rafts (7), which may serve to sort receptor molecules, although this function still needs to be formally proved *in vivo*.

The hydrolysis of sphingomyelin results in the formation of ceramide, which is an amide ester of D-erythro-sphingosine and a fatty acid containing 2-32 (or even longer) carbon atoms in the acyl chain (5, 6). The hydrolysis of ceramide is mediated by acid, neutral or alkaline sphingomyelinases. However, ceramide can be also generated by the *de novo* synthase pathway, a retrograde production from sphingosine *via* ceramidases and upon hydrolysis of complex glycosylated lipids or dephosphorylation of ceramide 1-phosphate (5, 9–11). The generation of ceramide from sphingomyelin in small rafts has a dramatic effect on the biophysical properties of these small membrane domains: Ceramide molecules are very hydrophobic and thereby these molecules have the tendency to spontaneously self-associate to small microdomains that further fuse to large ceramide-enriched membrane domains, also named platforms (5, 6, 12, 13). A variety of studies has shown the formation of such ceramide-enriched membrane domains in particular upon application of stress and stimulation of certain receptors, for instance upon infection of mammalian cells with pathogens such as *Pseudomonas aeruginosa*, *Neisseriae gonorrhoeae*, Rhinovirus or SARS-CoV-2, stimulation of receptors such as CD95, CD40 or DR5 or application of stress stimuli such as platelet activating factor, irradiation, UV-light or Cu^2+^ application (14–26). Although ceramide-enriched membrane domains do not seem to have specific signaling functions for all of these diverse stimuli, they are central for cell activation, because they serve to trap, cluster and organize receptor and intracellular signaling molecules in the cell membrane, such as CD95, CD40, DR5, β1-integrin or NADPH-oxidase (12, 19, 20, 23, 25, 27). The reorganization process of receptors and signaling molecules results in the spatial and temporal organization of the signalosome generated by a specific receptor or stress stimulus. This re-organization process allows receptors to associate with down-stream signaling molecules, to exclude inhibitory molecules, to concentrate a high number of receptor molecules in a small area of the plasma membrane and thereby to greatly amplify the primary signals finally permitting a specific receptor to transmit its signal into the cell (27). This mechanism is, in particular, mediated by the acid sphingomyelinase, which resides in lysosomes. Upon cellular activation, the acid sphingomyelinase is often exposed onto the cell surface by fusion of secretory lysosomes with the plasma membrane (12, 28, 29). This fusion process of secretory lysosomes with the plasma membrane is mediated by a syntaxin 4-regulated transport of intracellular vesicles (28), although many details of this process are still unknown. The acid sphingomyelinase generates ceramide on the cell surface, for instance after irradiation or CD95 stimulation (12, 29), but it is also possible that ceramide is already generated in secretory lysosomes and exposed together with the acid sphingomyelinase. These processes are certainly not exclusive and a local surface acid sphingomyelinase activity may lead to further formation of ceramide and thereby control the strength and the duration of signals generated by receptor clustering.

In addition to re-organizing the cell membrane and to form membrane platforms, ceramide has been also shown to directly regulate several molecules, i.e. cathepsin D (30), phospholipase A_2_ (31), kinase suppressor of Ras (32), ceramide-activated protein serine-threonine phosphatases [CAPP] (33), protein kinase C isoforms (34, 35), phosphatase 2A (36), Lc3B (37) and phospholipase D (38). Studies by Schneider-Schaulies et al. demonstrated an important impact of ceramide on the cytoskeleton, in particular F-actin, ezrin and moesin, and migratory properties of lymphocytes (39), although it is unknown whether this is mediated by a direct interaction of membrane ceramide with cytoskeletal proteins or *via* an indirect effect of ceramide. Further, it has been demonstrated that ceramide regulates the activity of ion channels, such as the potassium channel Kv1.3 and calcium release-activated calcium channels (40–43), but it is again unknown whether this effect is direct or mediated *via* re-organization and/or lipid-protein interactions of the ion channels in ceramide-enriched membrane domains.

## 3 Thyroid eye disease

### 3.1 Clinics

Graves’ disease (GD) is an autoimmune disorder that is caused by autoantibodies against the thyroid stimulating hormone receptor (TSHR). These anti-TSHR-antibodies are often stimulating antibodies of the TSHR and therefore lead to an overstimulation of the thyroid gland (44). TSHR-stimulating autoantibodies bind to the TSHR at a similar extracellular site as the thyroid stimulating hormone (TSH) itself and cause deregulated TSHR hyper-activation (44) resulting in Graves’ disease (45).

The continuous stimulation of TSHR leads to uncontrolled production of thyroid hormones T3 and T4 in the thyroid. To date, TSHR hyper-activation is treated by anti-thyroid drugs correcting the “clinically evident” consequences. This involves pharmacological downstream suppression of the enzyme thyroperoxidase to impair thyroid hormone synthesis and thyroid hormone release from the thyroid. About half of the patients go into remission over time due to spontaneous decay of anti-TSHR antibodies, the other half suffers from chronic relapsing course due to persistence of the immune response (46). In these patients the thyroid will be treated by thyroidectomy or radioiodine therapy. In some of these patients anti-TSHR antibodies persist particularly after radioiodine therapy, and therefore extrathyroidal manifestations can also occur later in the course of these cases with Graves’ disease (47). The most common extrathyroidal manifestation is thyroid eye disease, also known as Graves’ Orbitopathy (TED or GO) (46, 48, 49). Acropachy and pretiabial myxedema are much less common. About half of Graves’ disease patients develop thyroid eye disease and 3 to 5% develop a severe course of disease threatening their vision (46).

### 3.2 Molecular mechanisms of thyroid eye disease

The TSHR belongs to the glycoprotein hormone receptors, a subfamily of class A G-protein-coupled receptors. The interplay between TSH and TSHR constitutes the pituitary-thyroid axis, which tightly regulates growth, development and metabolism. There have been numerous publications showing the relation between anti-TSHR antibodies level and activity and thyroid eye disease (e.g. 50–56).

Anti-TSHR antibodies do not only bind to follicular epithelial cells of the thyroid gland, but also to retro-orbital fibroblasts, which express TSHR (57). Therefore, autoimmune anti-TSHR autoantibodies activate the TSHR in the orbit causing Thyroid eye disease, a potentially quality of life-reducing and sight-threatening disease.

Binding of anti-TSHR antibodies to the TSHR results in direct activation of target cells, but also immediate stimulation of hyaluronan secretion, one of the central patho-mechanisms of thyroid eye disease, since hyaluronic acid binds water molecules, which leads to increase of volume of orbital tissues (58).

Anti-TSHR antibodies also stimulate human orbital preadipocytes to differentiate into mature adipocytes *via* phosphoinositide 3-kinase activation, which also contributes to the tissue volume increase in the orbit (59, 60). These two pathomechanisms lead clinically to swollen extraocular muscles and exophthalmos. Since the volume increases occur in a bony-limited orbit, it can lead to more or less compression of the orbit depending on the strength of the orbital ligaments, impairment of orbital venous and lymphatic outflow and tissue hypoxia (61, 62).

In addition to the stimulation of TSHR in fibroblasts and adipocytes, the autoimmune reaction in the orbit also leads to infiltration with macrophages and T cells into orbital tissues, resulting in the release of several potent inflammatory mediators. In the initial phase of the disease an increased activity of Th1 lymphocytes has been shown, which leads to the production and release of typical cytokines, such as IL-1β, IL-2, TNF-α and IFN-γ, enhancing the proliferation of orbital fibroblasts and production of glycosaminoglycans (63). These cytokines further attract infiltration of immune cells T and B cells, monocytes, and neutrophils *via* cytokines like CCL2, CCL5 and CCL20, which migrate into the orbital tissue and enhance the inflammatory process (64, 65). During the ongoing inflammatory process Th2 lymphocytes get activated as well and further release cytokines, such as IL-4, IL-5, IL-10 and IL-13 (66). In the later stages of the disease tissue expansion, remodeling and fibrosis dominate the autoimmune disorder (67). In addition, an impaired suppressor function of Treg lymphocytes in patients with thyroid eye disease has been suggested (65). Tregs are important for tolerance to self-antigens and for modulating the autoimmune system by the release of anti-inflammatory cytokines (68). Patients with thyroid eye disease show a significant increase in Th17-cells in the serum, contributing to the inflammatory infiltration by mediating pro-inflammatory responses (69). This suggests a dysregulation of the Th17/Treg balance towards lower Tregs and higher Th17 levels in thyroid eye disease patients.

In addition, sphingolipids are known to be involved in the control of development, differentiation, activation, proliferation and attraction of lymphocytes (70–72).

These changes are found to varying degrees in human tissue and also in the thyroid eye disease animal model. This high variability of autoimmune-driven inflammation in *in vivo* and *in vitro* models is also reflected by the clinical manifestation, which varies greatly among patients depending on their age and risk factors.

In the past, therapeutic efforts focused on anti-inflammatory therapy with the aim to stop the inflammatory reaction in the orbit and the autoimmune reaction in general (73). In 2017, the results of a phase 2 study were published, in which for the first time patients with thyroid eye disease were treated with an antibody that blocks the IGF1R (74). The results mark a turning point of thyroid eye disease treatment, because for the first time, exophthalmos (80%) and motility impairment (approx. 50%) could be efficiently remedied (75). However, first case reports for the IGF-1R antibody therapy show recurrences after this therapy, since the autoimmune reaction does not seem to be influenced by this therapy (76). Therefore there is a strong need to further elucidate the autoimmune reaction in thyroid eye disease to develop causative treatments. Sphingolipids may play an important role here.

## 4 Sphingolipids in thyroid eye disease

The role of sphingolipids in thyroid eye disease is largely unknown. Studies by our group demonstrated that fibroblasts in the orbit of patients with thyroid eye disease exhibit an altered phenotype with the expression of CD40, which is a co-stimulatory receptor usually expressed on the surface of immune cells such as B-lymphocytes or antigen-presenting cells (for review see e.g. 77). Activated T lymphocytes express the ligand of CD40, i.e. CD154. Binding of CD154 to its cognate receptor CD40 results in B lymphocyte activation, in particular immunoglobulin class switching from IgM to IgG (77). The T cell mediated help is a central step in immune stimulation in general and also plays an important role in the pathogenesis of autoimmune disorders (77). Moreover, expression of CD40 is not restricted to B-lymphocytes and antigen presenting cells, but it is also present on fibroblasts of the orbit in patients with thyroid eye disease (78–80). Here, the expression of CD40 in the diseased orbit creates a link to previous studies demonstrating that cellular stimulation *via* CD40 results in a rapid activation of the acid sphingomyelinase, a translocation of the acid sphingomyelinase onto the cell surface and the generation of surface ceramide with the subsequent formation of large ceramide-enriched membrane platforms that cluster and signal CD40 (19). Molecular studies demonstrated that the transmembranous domain of CD40 mediates clustering of CD40 (81). In these studies, the transmembranous domain of CD40 was exchanged with the transmembranous domain of CD45, which does not cluster in ceramide-enriched membrane domains (81). This chimeric fusion protein did not cluster in ceramide-enriched membrane domains anymore and did not signal under physiological conditions, while forced cross-linking and clustering of this chimeric molecule using crosslinked antibodies reconstituted CD40-typical signaling (81). This establishes a central role of ceramide-mediated clustering of CD40 for signaling of CD40.

Consistent with the hypothesis that CD40 clustering also mediates activation of orbital fibroblasts in thyroid eye disease, we observed an activation of the acid sphingomyelinase and a release of ceramide upon activation of CD40 in thyroid eye disease-derived fibroblasts (80).

The generation of ceramide also allows increased formation of sphingosine 1-phosphate and it was demonstrated that activation of fibroblasts isolated from the orbit of patients with thyroid eye disease also shows a higher stimulation of sphingosine kinase 1 (80), which converts sphingosine to sphingosine 1-phosphate (82). CD40 stimulation of these fibroblasts resulted in a further increased formation of sphingosine and a release of sphingosine 1-phosphate (80). Sphingosine 1-phosphate is known to be central in the regulation of T lymphocyte emigration into tissues (82). Several studies (72, 83, 84) demonstrated that sphingosine 1-phosphate is important in key aspects of thyroid eye disease, including the induction of inflammation, increased adipogenesis in the orbit and the induction of orbital fibrosis (72, 83, 84). These studies demonstrated increased expression of sphingosine 1-phosphate receptors 1, 2 and 3 in the orbit from patients with thyroid eye disease (72, 83, 84). *In vitro* data showed that adipocytes responded with increased differentiation upon stimulation with sphingosine 1-phosphate, which was prevented by an inhibitor of the sphingosine 1-phosphate receptor 1 (72). Further, orbital fibroblasts from patients with Graves’ orbitopathy exhibited an increased production of reactive oxygen species, which was also blocked by an inhibitor of sphingosine 1-phosphate receptors (83). It remains to be determined whether the increased production of reactive oxygen species also mediates a constitutive activation of the acid sphingomyelinase, which is regulated by redox mechanisms (85, 86). Such a stimulation would also result in the formation of ceramide-enriched membrane platforms that serve to cluster NADPH-oxidase and thereby further promote the formation of reactive oxygen species (86). Such a vicious cycle, once initiated by the inflammation-triggered alteration of orbital fibroblasts may continuously drive the autoimmune disease, even after down-regulating antigen expression. It is also tempting to speculate that cigarette smoking, one of the most important risks for thyroid eye disease (87), drives such a reactive oxygen species – acid sphingomyelinase – ceramide cycle and thereby promotes the disease. In accordance, Ko et al. demonstrated increased expression levels of sphingosine 1-phosphate 1 receptor mRNA and of sphingosine 1-phosphate in orbital fibroblasts from patients with GD, in particular after stimulation with cigarette smoke extract (84). The changes of sphingosine 1-phosphate correlated with increased formation of collagen Iα, fibronectin, and α-smooth muscle actin, as well as IL-1β–induced expression of metalloproteases MMP-1, MMP-2, MMP-9, and TIMP-1, events that were prevented by inhibition of sphingosine 1-phosphate receptors (83).

Our own studies demonstrated that stimulation of fibroblasts isolated from the orbit of patients with Graves’ orbitopathy *via* CD40 attracted T lymphocytes, which was blocked by a sphingosine kinase inhibitor (80). Thus, alterations of fibroblasts in the orbit of patients with Graves’ orbitopathy may drive the influx of T lymphocytes into the orbit *via* the generation of sphingosine 1-phosphate.

Collectively, these studies indicate that sphingosine 1-phosphate seems to be important for several aspects of thyroid eye disease, in particular adipogenesis and activation of local fibroblasts. At present, the role of ceramide and sphingosine in thyroid eye disease requires definition.

## 5 Sphingolipids in autoimmune lymphocyte activation

The state of the current knowledge on the role of sphingolipids in thyroid eye disease is presented above. However, there is good evidence to postulate an important role of the acid sphingomyelinase in autoimmune disorders. Thus, it was shown that the acid sphingomyelinase plays an important role in multiple sclerosis (1, 2, 4), the most important autoimmune disorder of the central nervous system and the leading cause of neurological disability among young adults in the Western world (88, 89). Multiple sclerosis is characterized by focal inflammation in the central nervous system eventually leading to local demyelination, loss of neurons, glia scars and neurological symptoms, such as motor and sensory deficits or impairment of vision. As in thyroid eye disease the autoimmune process is initiated by infiltration of CD4^+^ and CD8^+^ T lymphocytes as well as macrophages (88, 90–92), which then orchestrate and execute central nervous inflammation and damage. Studies on the role of the acid sphingomyelinase in multiple sclerosis demonstrated that genetic deficiency of acid sphingomyelinase prevented many aspects of multiple sclerosis, including blood-brain barrier disruption, influx of immune cells into the central nervous system and, thereby, neuroinflammation (1, 2). Acid sphingomyelinase-deficient mice were almost completely protected against the induction of an experimental multiple sclerosis, i.e. experimental autoimmune encephalomyelitis (1, 2). Experimental autoimmune encephalomyelitis was also inhibited by treatment of the mice with functional inhibitors of the acid sphingomyelinase, such as amitriptyline or sertraline (2). These drugs interfere with binding of the acid sphingomyelinase to membranes and displace the acid sphingomyelinase from intralysosomal membranes resulting in proteolytic degradation of the acid sphingomyelinase (93–95). These drugs are often antidepressants (94, 95).

Deficiency or pharmacological blockade of the acid sphingomyelinase also prevented the induction of another autoimmune disorder, i.e. autoimmune arthritis (3). These studies revealed an induction of severe arthritis upon autoimmune immunization, which was prevented by genetic deficiency or pharmacological inhibition of the acid sphingomyelinase (3). In summary, the data clearly demonstrate that the acid sphingomyelinase plays a pivotal role in autoimmune disorders, although the molecular mechanisms of the involvement of the acid sphingomyelinase are still unknown. It is therefore tempting to speculate that the acid sphingomyelinase also plays a role in GD, although this needs to be tested.

Sphingomyelinases have been shown to regulate several aspects of lymphocyte biology, although a comprehensive picture is missing.

Several studies have shown that deletion of the acid sphingomyelinase results in an increased formation of regulatory T lymphocytes that are able to down-regulate or even inhibit immune responses (96, 97). Acid sphingomyelinase-deficient mice have higher number of regulatory T cells compared to littermate control mice. *In vitro* stimulation of acid sphingomyelinase-deficient T-lymphocytes resulted in higher number of Foxp3+ -induced regulatory T cells, compared with control T-cells (97). This effect might be mediated by Akt and Rictor, since acid sphingomyelinase-deficient induced regulatory T cells demonstrated reduced phosphorylation of Akt and reduced expression of Rictor, a protein that complexes with mTor2 and has been shown to be involved in regulation of cell growth and proliferation (96).

The generation of regulatory T cells in mice lacking the acid sphingomyelinase might explain, at least in part, the inhibition of autoimmune responses in acid sphingomyelinase-deficient mice or upon treatment with pharmacological inhibitors of the acid sphingomyelinase.

A recent study using T cell-specific acid sphingomyelinase- or acid ceramidase-deficient mice revealed that T cell-specific ablation of acid sphingomyelinase resulted in reduced ceramide levels in T cells and an impairment of T cell responses, whereas T cell specific deletion of the acid ceramidase elevated T cell activation (98). Decreased ceramide concentrations promoted differentiation of CD4^+^ regulatory T cells, but also negatively interfered with cytotoxic activity of CD8^+^ T cells, which was improved by elevated ceramide concentrations (98). The studies also linked the T cell receptor (TCR/CD3) complex to ceramide-enriched membrane domains, since ceramide co-localized with the TCR/CD3 complex forming an immune synapse in stimulated T cells, an event that seems to regulate the strength of TCR/CD3 signaling (99).

Several studies imply that also neutral sphingomyelinases are important in T cell activation (100, 101). These studies demonstrated that inhibition of neutral sphingomyelinases interfered with lymph node homing and adhesion of T cells to activated endothelial cells (99, 100). In accordance, pharmacological or genetic ablation of neutral sphingomyelinase 2 interfered with T cell polarization, suggesting that neutral sphingomyelinases are involved in T cell recruitment and migration (99, 100). Besides regulation of T cell polarization and migration, neutral sphingomyelinase-2 is also critically involved in fine-tuning of signaling generated *via* the TCR/CD3 (99, 100). Thus, deletion of neutral sphingomyelinase-2 resulted in hyper-responsivity of T cells upon stimulation *via* CD3/CD28 (99, 100). This effect seems to be mediated by an increased metabolic activity with an accumulation of ATP in mitochondria and higher basal glycolytic activity in lymphocytes upon downregulation of neutral sphingomyelinase-2 (101). Whether down-regulation of neutral sphingomyelinase-2 results in an altered immune response *in vivo* remains to be determined.

## 6 Perspective

**Figure 3:**
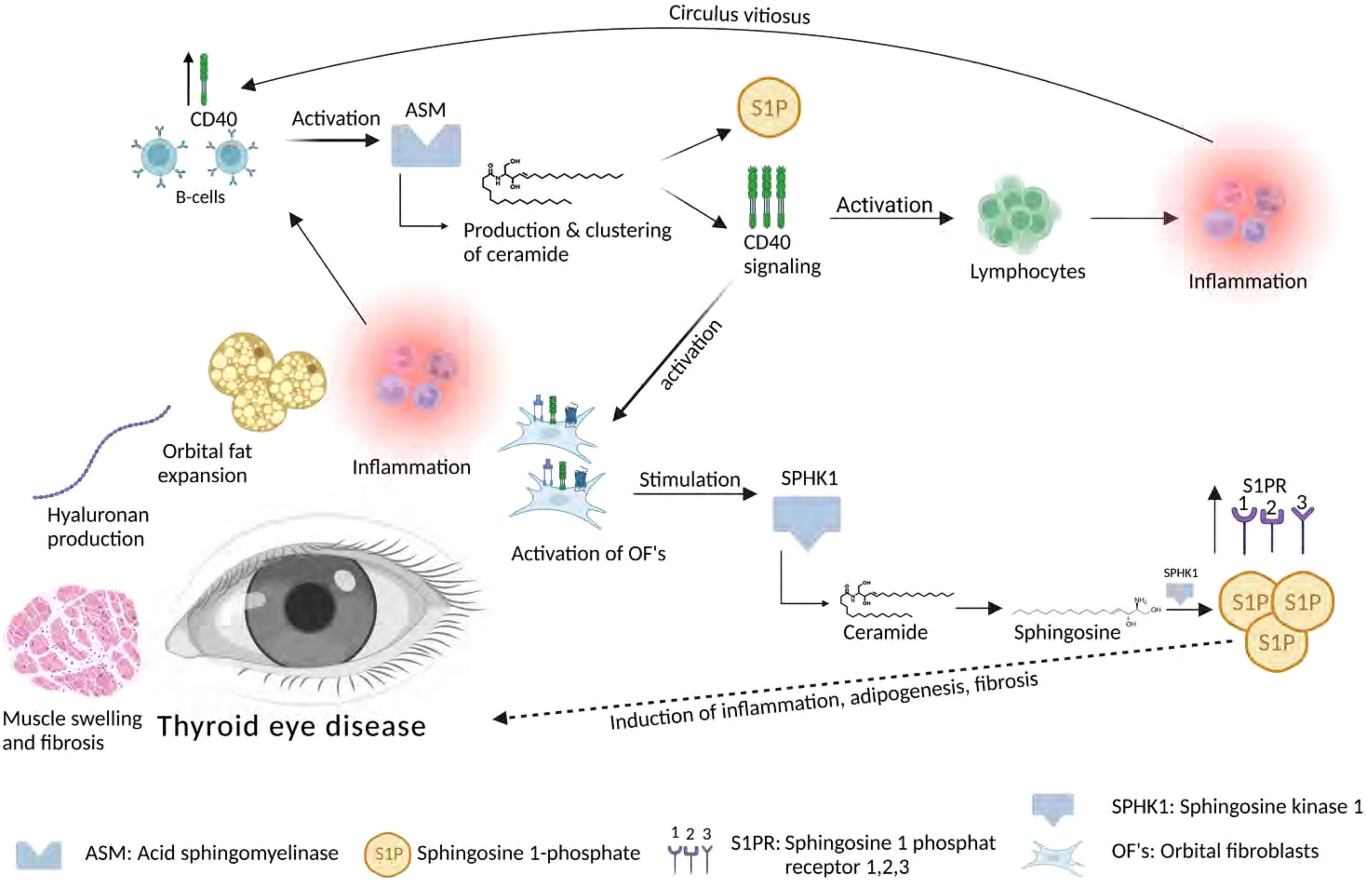
Possible function of sphingolipids in thyroid eye disease (TED).

The role of sphingolipids in thyroid eye disease, in particular sphingomyelin, ceramide, sphingosine and sphingosine 1-phosphate and of the enzymes involved in the metabolism of these sphingolipids such as acid sphingomyelinase, acid ceramidase and sphingosine kinases requires definition and only a few data are available. An overview is presented in **Figure 3**. However, already these few data clearly indicate that this pathway and in particular the acid sphingomyelinase and ceramide have a very important role in the immune-pathogenesis of autoimmune disorders such as multiple sclerosis, immune arthritis and possibly also thyroid eye disease. Further, sphingolipids, in particular ceramide and sphingosine 1-phosphate seem to be critically involved in regulating and mediating the local alterations of the orbit in thyroid eye disease. Therefore, it might be very interesting to test the development of thyroid eye disease in animal models and to investigate the impact of a genetic deficiency or a pharmacological inhibition of the acid sphingomyelinase on thyroid eye disease. These studies may also serve to develop novel treatments of thyroid eye disease based on inhibition of the acid sphingomyelinase and/or neutralization of ceramide and sphingosine 1-phosphate.

## Author contributions

The authors wrote the manuscript together and all authors contributed to writing and editing.
